# A retrospective autopsy study of histopathologic spectrum and etiologic trend of fulminant hepatic failure from north India

**DOI:** 10.1186/1746-1596-2-27

**Published:** 2007-07-27

**Authors:** Prasenjit Das, Deepali Jain, Ashim Das

**Affiliations:** 1 Department of Histopathology, Postgraduate Institute of Medical Education and Research, Chandigarh, India

## Abstract

**Background:**

Fulminant hepatic failure (FHF) is rapidly fatal and liver transplant is the treatment of choice. The condition is known for its heterogeneity of defining criteria, clinical presentation, histologic spectrum and etiologic factors. The etiology of FHF varies widely, some of which includes viral hepatitis, drug overdose and idiosyncratic drug reactions. The identification of the etiology of FHF is critically important, because it influences the management. A histopathological classification of FHF has not been reported earlier in the literature.

**Methods:**

The current study was conducted retrospectively on 224 autopsies at a tertiary care hospital in India. In all of these cases the liver was examined grossly and microscopically. Clinical findings, serological data and immunohistochemical findings were correlated with the morphological subtypes and a consensus morphological classification was formulated.

**Results:**

Young females, especially those in the reproductive age group were most susceptible to the disease. Hepatotropic viruses and drugs were the likely causes in most of the patients. Clinical presentation is important, as delayed onset of encephalopathy or the subacute FHFs lead to maximum mortality. After careful gross and microscopic examination the morphological findings of FHF were divided into four distinct categories. Histologic typing can sometimes be misleading if solely made on H & E slides without application of special stains.

**Conclusion:**

Fulminant hepatic failure is a medical emergency, proper histological categorization can help in deciding the treatment modalities.

## Background

Fulminant hepatic failure (FHF) is a rapidly fatal condition when untreated. It is defined as the rapid development of hepatocellular dysfunction and encephalopathy in a person with no prior history of liver diseases [1]. Till now, this condition was described in different ways by various authors. In 1970, the term FHF was coined to describe the onset of encephalopathy within 8 weeks of the first evidence of jaundice without any pre-existing liver disease [2]. In mid- 80s, French investigators divided FHF into fulminant (encephalopathy within 2 weeks of first appearance of jaundice) and subfulminant (encephalopathy between 2 and 12 weeks) [3]. More recently, a further classification into hyperacute (encephalopathy within a week), acute (1 to 4 weeks) and subacute (5 to 26 weeks) FHF has been made [1,4]. These classifications are based on clinical presentation of the patient, however histopathologic spectrum of various subtypes of FHF has not been classified yet [5,6].

The etiological factors of FHF are diverse in different geographical areas. In United States some investigators documented viral hepatitis as the leading cause of FHF (in one eighth of total FHF cases) followed by drug toxicity and idiosyncrasy, while other investigators found acetaminophen toxicity followed by non A non B non C hepatitis as the leading cause of FHF [7-9]. Etiology also differs between different age groups. Acute hepatitis A is the commonest cause in childhood, and has the best prognosis. Hepatitis B, C, and D rarely cause fulminant hepatic failure in childhood while hepatitis E virus may be associated with fulminant hepatic failure in children living in Indian subcontinent [10,11]. Consideration of a possible less common etiology, such as lymphoma or obesity is of considerable clinical importance and may be needed to know specific treatment measures [3,12].

Though there is no specific therapy for FHF, liver transplantation is recommended for situations in which spontaneous recovery appears unlikely [13]. In literature it has been documented that 40% of patients with FHF survives without liver transplantation [14]. A careful and individualistic approach is needed when treating an FHF patient. A detailed clinical, etiologic and histopathologic correlation is required. The current study on FHF therefore was conducted retrospectively on autopsy material from a single tertiary care hospital in North India, to identify the histopathologic spectrum and to determine the etiologies if possible.

## Methods

A retrospective study was performed on two hundred twenty four autopsy cases of fulminant hepatic failure since 1983 to 2002 (20 years) from our archive in Postgraduate Institute of Medical Education and Research, Chandigarh, India. We included these cases in our study depending on the history of rapid development of hepatocellular dysfunction in the form of jaundice, coagulopathy or presence of encephalopathy without prior history of any liver disease. The cases of rapid development of liver dysfunction with previous history of liver diseases (e.g. cirrhosis, Wilson's disease or hemochromatosis) or histologically proven hepatic necrosis without clinical evidence of encephalopathy and in which no clinical diagnosis was made, were excluded from the study. A detailed gross and microscopic examination was carried out. Gross weight, architecture, capsular surface, cut surface and portal vasculature of liver were studied carefully. At least three Hematoxylin & Eosin stained slides along with special stains like Gordon and Sweet's reticulin, Masson trichrome, Shikata's orcein, Elastic Van Gieson, and Periodic acid Schiff with and without diastase were studied in every case. Immunohistochemistry for HBsAg and HBcAg was carried out in all the cases. Serological data for viral and autoimmune causes was retrieved. Wherever possible, important antemortem drug history was recorded. The four histological classes were defined as follows in our study:

1. **Extensive multiacinar confluent hepatic necrosis without regeneration **– Characterized by diffuse transacinar loss of hepatocytes and replacement of hepatocytes by acellular debri, fibrin and acute inflammatory infiltrate with absence or occasional presence of macrophages. No deposition of elastic fibers should be seen on special stain.

2. **Multiacinar confluent hepatic necrosis with regeneration **– Characterized by restricted parenchymal loss with associated regeneration. The regenerative nodules showed hepatocytes in double cords along with mild nucleomegaly and cholestasis along with varying degree of cholangiolar regeneration. Macrophages were the predominant population of inflammatory cells.

3. **Bridging hepatic necrosis with regeneration **– Characterized by narrow band of necrosis extending from central vein to portal tract or portal tract to portal tract. Number of inflammatory cells and macrophages varied in this type. Elastic fiber deposition may or may not be present.

4. **Differential pathology **– Characterized by admixture of the above mentioned pathologies in any extent. For e.g. confluent hepatic necrosis with or without regeneration in one area and bridging hepatic necrosis with extensive regeneration in the other areas.

## Results

During the period of twenty years, since 1983 to 2002, a total number of 10930 autopsies were performed; amongst which 224 of FHF cases were selected based on our inclusion criteria. Females were affected most, as a total of 170 females with FHF were identified in our study (75.8% of total number of FHF cases), which included 61 pregnant females, aged between 20 and 32 years. Forty-six deceased were male, aged between 13 to 63 years and eight were children below 12 years of age. In our study, 13 hyperacute and 48 acute forms of FHF were noted in pregnant females. In males and females not in reproductive age group, 53 hyperacute, 104 acute and 6 subacute FHFs were identified.

### Pregnancy with FHF (61 cases)

A total 423 maternal deaths were recorded during this period of time. Amongst which 95 deaths (22.4%) were related to liver diseases. Of these, 61 deaths (64.2% of liver related deaths) were due to fulminant hepatic failure. The pregnant females who died of FHF presented between 28^th ^to 38^th ^weeks of their pregnancy. Of these, in eight cases, hepatitis B virus infection was detected. In rest of the cases, etiology was not made antemortem or postmortem. Pregnancy with hepatitis E infection was not identified in the present study.

Clinically 13 of these cases had hyperacute presentation and rest of them presented with acute form. A total of 46 autopsies in pregnant females demonstrated multiacinar confluent hepatic necrosis without regeneration and in 12 cases multiacinar confluent hepatic necrosis with regeneration was noted.

### FHF in other patients (163 cases)

There were Forty-six male patients, aged between 13 to 63 years, eight children below 12 years of age and 105 females not in reproductive age group. Most common age groups affected were 13 and 29 years (61%), followed by 30–39 years (18%), 40–49 years (8%) and 50 years onwards (13%). According to the clinical presentation, 53 cases were hyperacute (49 females and 14 males), 104 cases were acute and 6 cases were of subacute FHF. On histologic examination, multifocal confluent hepatic necrosis without regeneration was the most common pattern (47.8%), followed by multifocal confluent hepatic necrosis with regeneration (26.9%), bridging hepatic necrosis (5.5%) and differential pathology in 30 cases (18.4%).

### Etiologic Factors

In a total number of 224 cases, etiology could be determined in 49 cases, including 8 pregnant females with HBsAg positivity. Etiologic factors in rest of the 41 cases were as follows: HBsAg positivity in 18 cases (58%), HCV Ab positivity in 4 cases (12%) and positive HEV serology in 8 cases. One case did show combined HBsAg and HEV positivity. None of the pregnant females in our study was HEV serology positive. In six cases (3%), drug toxicity was the offending agent and the drugs were INH (4 cases) and acetaminophen (2 cases). Primary hepatic lymphoma was identified in two cases and autoimmune hepatitis with FHF and ischemic FHF were identified in one case each. Rare causes like primary hepatic lymphoma, autoimmune hepatitis or ischemia showed extensive multiacinar confluent hepatic necrosis without regeneration. In rest of the cases, etiology could not be confirmed or it was unknown.

### Pathologic Findings

On gross examination, in massive confluent hepatic necrosis, normal size and shape of liver remained undisturbed. While in subacute or massive hepatic failure with formation of regenerative nodules - shrinkage of liver size, wrinkled opaque capsular surface, dark discoloured soft cut surface, concentration of the porto-venous radicals close to one another and bile stained regenerative nodules were identified. A bridging hepatic necrosis showed reticulated cut surface where the viable part of the lobules were seen separated by porto-portal or porto-central thin grey-white lines of necrosis.

Histologic subclassification was as follows:

### Extensive multiacinar confluent hepatic necrosis without regeneration – (Fig 1 &2)

**Figure 1:**
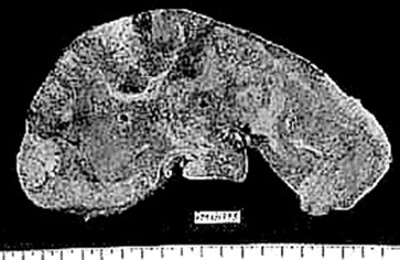
Gross photograph of a liver slice with massive confluent hepatic necrosis. Normal liver size and shape are maintained.

**Figure 2:**
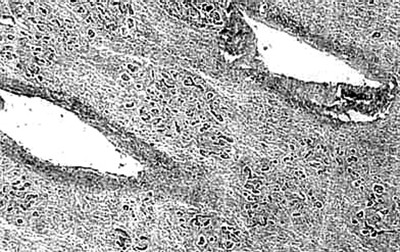
Photomicrograph of liver section showing multiacinar confluent hepatic necrosis. Condensation of the central veins are seen with scant macrophages, H & E, × 40.

There was transacinar extensive necrosis with maintained liver architecture. Acute inflammatory infiltrate with only occasional macrophages was identified.

This pattern was identified in 46 pregnant females and 78 other patients.

### Multiacinar confluent hepatic necrosis with regeneration – (Fig 3, 4, 5, 6)

**Figure 3:**
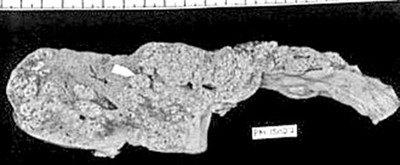
Gross photograph of liver with subacute hepatic failure showing multiple regenerative nodules. Size of the liver has reduced and the shape has altered.

**Figure 4:**
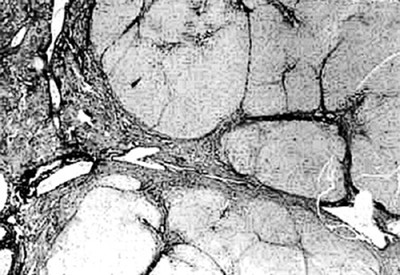
Photomicrograph of liver showing varying sized regenerative nodules surrounded by passive septae. This condition can be confused with cirrhosis by neophytes, H & E × 40.

**Figure 5:**
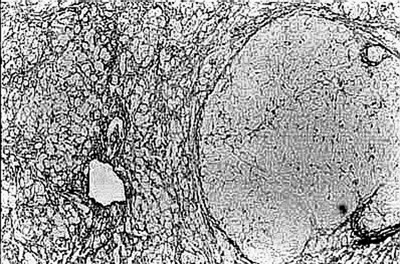
Silver impregnation technique highlightes the compressed hepatic reticulin archietecture, Gordon and Sweet's silver impregnation technique × 40.

**Figure 6:**
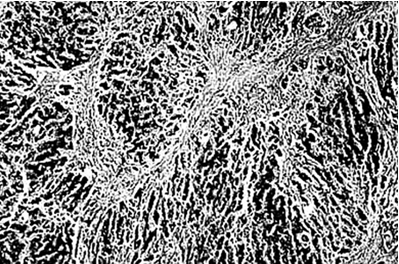
Shikata's orcein stain showed absence of elastic fibres in the passive septae, Shikata's orcein silver stain × 100.

There was transacinar extensive hepatocyte necrosis with formation of regenerative nodules at places. Due to formation of regenerative nodules, the reticulin framework of the necrotic area collapsed and formed 'passive septae'. The necrotic zone showed presence of many macrophages along with a mixed population of inflammatory cells. Additional difficulty we faced was differentiation of this group from cirrhosis grossly and on H & E sections, because the passive septae formed by hepatocytes, resembled fibrous bands. Shikata's orcein stain was helpful, as it did not show any elastic fiber deposition in the passive septae.

This pattern was identified in 12 Pregnant and in 44 other patients

### Bridging hepatic necrosis with regeneration – (Fig 7 &8)

**Figure 7:**
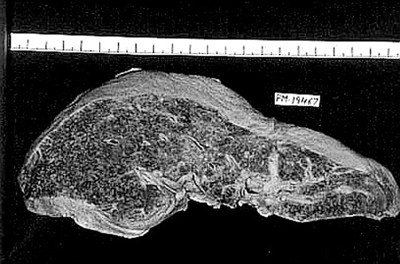
Gross photograph showing reticulated cut surface; liver capsule is opaque and corrugated.

**Figure 8:**
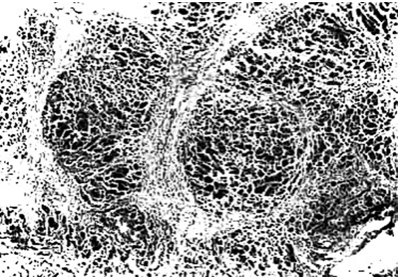
Photomicrograph of liver showing thin necrotic bands connecting portal tracts to portal tracts and central veins; inflammatory cells are scanty without collagen deposition, Masson trichrome × 40.

In these cases a typical reticulated cut surface of liver was identified. On microscopic examination narrow bands of necrosis were seen present between portal tract to central vein or another portal tract. Inflammatory cells were less in comparison to the above mentioned types and composed of variable number of macrophages.

In our study 2 pregnant females and 11 other deceased were found to have this morphological spectrum.

### Differential pathology – (Fig 9)

**Figure 9:**
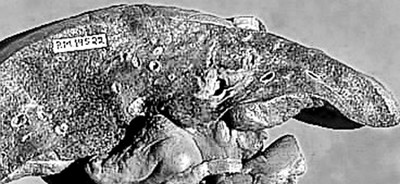
Gross photograph of liver with differential pathology. Central area of confluent hepatic necrosis flanked by areas with reticulated cut surface.

Admixture of the above mentioned pathologies in varying extent, both grossly and microscopically, was identified.

We noted this morphological variation in 1 pregnant and 30 other males and females

In the hyperacute FHF the histological pattern was multiacinar confluent hepatic necrosis. Multiacinar confluent necrosis, bridging hepatic necrosis and differential pathology were noted in both acute and subacute FHF. Multiacinar confluent hepatic necrosis with regeneration, bridging hepatic necrosis with regeneration and differential pathology were identified in subacute and chronic FHF. In the present study, the earliest change of regeneration was noted in three weeks.

## Discussion

Fulminant hepatic failure is characterized by the rapid onset of life threatening hepatic decompensation in patients who have no previous history of liver disease [1]. First step of management is triaging patients according to the presentation. Prognosis could be assumed from this triage. According to the present study, the most common presentation in FHF was acute (67.8%), followed by hyperacute (29.4%) type. Subacute FHF was rarest. Our findings were similar to a study from north India by Acharya et al [15], who found acute form of FHF was most prevalent. However these results do not correspond to the observations from United Kingdom, France and Japan, where the subacute type is the most common subtype reported [15]. The differences of clinical presentation between two geographical areas may be explained by the difference of etiologic agents, host factors, environmental factors and the nature of the study. Our study was a retrospective autopsy study, which may show different outcome from those studied on living subjects. In different published studies it has been shown that hyperacute liver failure has better survival rate (27%, 5 year survival) than acute (7%, 5 year survival) and subacute liver failures (0–1%, 5 year survival) [5,16]. A large number of female patients with FHF, in the present series, were pregnant and all of them were in third trimester of pregnancy.

During epidemics of HEV infection, pregnant women have been reported to have a high risk of developing acute hepatitis as well as FHF compared with females not in reproductive age and males [17]. However this was not substantiated in the current study; as we did not find any case of HEV infection in these females. Explanation for this difference is unclear. Based on the present findings it can be postulated that pregnant females in their third trimester with FHF have higher mortality than males with FHF. On contrary Acharya et al [15] did not find pregnancy per se or the duration of the gestation as predictors of the mortality in FHF.

The etiology of FHF is multifactorial, including viral hepatitis and drug toxicity [7], as can be seen in the present study where viral hepatitis is the major cause of FHF (79.5% of total known etiologies). Among the viruses, hepatitis B was responsible for 53% of FHF. In other series, hepatitis B was reported in around 50–60% cases of FHF [18]. Overall, hepatitis B is reported as the most common cause of FHF worldwide [8]. Special virological factors may predispose to FHF in hepatitis B. These factors could include a role of mutant strain, coinfection or superinfection with the delta (hepatitis D) virus, and chemotherapy related reactivation of hepatitis B virus [19]. However observations of studies from Indian subcontinent incriminate non-A, non-B virus as the major cause of FHF in this country [15,20]. Since in the current study etiology could be determined in only 49 cases, therefore in rest of the cases possibility of seronegative hepatitis can not be completely ruled out. Only one case in this series was positive for Hepatitis A serology. In literature, one case of FHF with proven hepatitis A has been documented previously from India [21]. Most studies reviewed indicate that hepatitis C virus does not result in a fulminant course [18], whilst 8.1 % of our FHF cases showed positive hepatitis C serology. Two reports from France and Japan [22], noted coinfection of hepatitis C and hepatitis B virus in upto 50% patients with fulminant hepatic failure but it was not observed in our cases. Regarding hepatitis E, the virus usually shows a fulminant course in pregnant women in Indian subcontinent, while it plays a minor role in the west and is mainly reported in travelers to endemic areas [11,23]. HEV in the present study was the second most common cause (16.3%) for FHF after hepatitis B (53%) infection in males and females of nonreproductive age group. Most cases of drug related FHF resulted from acetaminophen overdose. In fact, acetaminophen is the most common cause of FHF in United States [9,14]. This drug is directly hepatotoxic and predictably produces hepatocellular necrosis with overdose [24]. Numerous other drugs including halothane, Isoniazid, Sodium Valproate, Carbamazepine, and Phenytoin have been implicated as etiological factors in FHF [3,10]. In the present study, acetaminophen (two cases) and Isoniazid (four cases) were the two offender drugs responsible for FHF. Drugs were found next to viruses as etiological factors.

In all reported series of FHF, in a sizable number of cases (10% to 45% in the west) no etiology can be demonstrated [25]. In the present study, out of 224 cases studied, in only 49 patients (21.8%) etiologies could be confirmed. In rest of the cases, etiology could not be determined or was unknown. Our study is a retrospective study and the unavailability of the serological facilities in the past may have contributed for the same.

A group of rare etiologies are worth considering because they may be curable with specific therapies. One of such factors is obesity. Since our study is retrospective and BMI of patients were not documented, we could not evaluate obesity as an etiologic factor in our patients. Canbay et al did a retrospective study on 34 patients of acute on chronic liver failure and acute liver failure, and concluded that elevated BMI coincided with a higher rate of acute on chronic liver failure rather than with acute liver failure [12].

**Figure 10:**
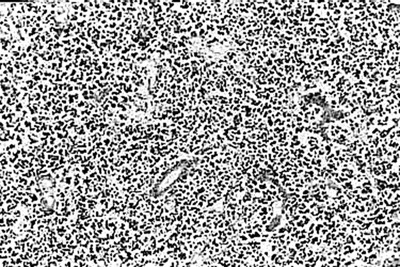
Photomicrograph showing diffuse destruction of the liver archietecture by infiltrating malignant lymphoid cells, H & E × 40.

Other such factors are hepatic lymphoma (Fig 10), autoimmune hepatitis and ischemic hepatic failure. Recently Mudawi and Yousif [26] have reported association of FHF with non Hodgkin's lymphoma. Interestingly, in NHL, chemotherapy induced FHF has also been described in the literature, however in our case no such history was detected. FHF traditionally is associated with up to 90% mortality. Earlier recognition of the causative agents and improved management has led to an improved survival. However unfortunately, a considerable number of patients succumb to this disease [27].

The gross findings of FHF are not always informative enough for proper morphological classification or in determining the cause. However, the overall size, weight of the liver and features on cut surface are important to estimate the onset and the severity of the event. Liver size usually remains normal in hyperacute and acute FHF and massive hepatic necrosis without regeneration. On the contrary, the liver size shrinks when the sequence of events is relatively slower to start with or when associated with early events of regeneration. Clinically subacute liver failure and grossly shrunken liver size have been identified as a most frequent association with death in FHF [5,10,28].

The heterogeneity of morphological findings in FHF have been mentioned in previous studies by Hanau et al [5] and Shimizu et al [6], but not classified yet. Pathologists and hepatologists working in this field should be aware of the various morphological spectrums that can be seen in this condition, because morphological categorization can give an idea about possible outcome. An urgent percutaneous needle biopsy in FHF patients can deliver important information. Main histological subgroups identified in these 224 cases are: extensive multiacinar confluent hepatic necrosis without regeneration (126 cases), multiacinar confluent hepatic necrosis with regeneration (56 cases), bridging hepatic necrosis with regeneration (11 cases) and differential pathology (combination of other categories) (31 cases). The classification was based on the extent of the acinar hepatocyte destruction, pattern of necrosis, type of inflammatory cell infiltration, extent of deposition of elastic fibers (with Shikata's orcein) and presence of features of regeneration [5,28-30].

However diagnostic pitfalls in FHF should always be kept in mind to avoid determination of erroneous categorization. One of such problems is to differentiate the multiacinar hepatic necrosis with regenerative nodule from cirrhosis grossly or on microscopy. The passive septae formed by collapsed reticulin framework due to necrosis can mimic fibrous bands of cirrhosis. These collapsed bands are formed passively and not by active laying down of collagen fibers. Shikata's orcein stain should always be used routinely. Negative staining in such a condition determines passive septae formation. Determination of etiology is very difficult when liver has undergone necrosis. Immunohistochemistry in this condition can lead to erroneous diagnosis due to extensive background staining. This limitation was realized by other authors also [6,28,31]. That is why in a large percentage of FHF cases, etiology could not be ascertained. Moreover, serology facility was limited in past, hence we could not rule out the true idiopathic cases. It has been previously described that death rate is maximum in subacute hepatic failure [6,15]. From our morphological study it was noted that subacute FHF had the maximum number of macrophages within the necrotic zone, though it is not proved from this study, it can be assumed that the cytokines released by the accumulating macrophages produce systemic inflammatory response and multiorgan failure. Thus, we again emphasize the importance of proper histological classification, if it can be achieved on premortem needle biopsies. This information may help the surgeons to take urgent decision about the necessity of liver transplant in patients of subacute FHF.

Discussion of the treatment part is far from the scope of this study, however we feel to conclude the discussion with the emphasis on proper selection of patients for liver transplant for better outcome. On average 50% of FHF patients undergo liver transplant as liver transplantation is the most promising treatment.

## Conclusion

To summarize the important facts about FHF we could gather from this study, are:

• Morphologically FHFs may be categorized into four classes on premortem needle biopsies and transplant decision may be taken accordingly, as transplant is most needed and successful in subacute FHF.

• Submassive hepatic necrosis with nodular regeneration should not be confused with cirrhosis.

• Females, especially those in the reproductive age group are at risk, as 123 females including pregnant ones, of a total of 224 cases, died of FHF (54.91%). Pregnant females are affected most in third trimester.

• Pregnancy with FHF imparts a potential threat for maternal death, as 61 of 95 (64.2%) liver related maternal deaths were due to FHF.

• Most common antemortem-presentation was acute, followed by hyperacute and subacute FHFs. It can be assumed retrospectively that patient who presented with encephalopathy after 7 days of onset of jaundice or coagulopathy, die most frequently.

• Autoimmune hepatitis, primary hepatic lymphoma and ischemic hepatocyte necrosis can present with FHF.

## Competing interests

The author(s) declare that they have no competing interests.

## Authors' contributions

Each author contributed in the research performed to complete this report. PD and DJ wrote the majority of the article and also performed the data analysis. AD had overall responsibility for design, development, and conduct of the project. All authors read and approved of the final manuscript.
